# The mechanism of pollination drop withdrawal in *Ginkgo biloba* L.

**DOI:** 10.1186/1471-2229-12-59

**Published:** 2012-05-01

**Authors:** Biao Jin, Lei Zhang, Yan Lu, Di Wang, Xiao X Jiang, Min Zhang, Li Wang

**Affiliations:** 1 College of Horticulture and Plant Protection, Yangzhou University, Yangzhou, Jiangsu, 225009, China; 2 Key Laboratory of Plant Molecular Physiology, Institute of Botany, Chinese Academy of Sciences, Beijing, 100093, China

## Abstract

**Background:**

The pollination drop (PD) is a characteristic feature of many wind-pollinated gymnosperms. Although accumulating evidence shows that the PD plays a critical role in the pollination process, the mechanism of PD withdrawal is still unclear. Here, we carefully observed the PD withdrawal process and investigated the underlying mechanism of PD withdrawal, which will aid the understanding of wind-pollination efficiency in gymnosperms.

**Results:**

In *Ginkgo biloba*, PDs were secreted on the micropyle during the pollination period and persisted for about 240 h when not pollinated under laboratory conditions. The withdrawal of an isolated PD required only 1 h for evaporation, much less than a PD on the living ovule, which required 100 h. When pollinated with viable pollen, PDs withdrew rapidly within 4 h. In contrast, nonviable pollen and acetone-treated pollen did not cause PD withdrawal. Although 100% relative humidity significantly inhibited PD withdrawal, pollinated PDs still could withdraw completely within 48 h. Pollen grains of *Cycas revoluta*, which are similar to those of *G. biloba*, could induce PD withdrawal more rapidly than those of two distantly related gymnosperms (*Pinus thunbergii* and *Abies firma*) or two angiosperms (*Paeonia suffruticosa* and *Orychophragmus violaceus*). Furthermore, pollen of *G. biloba* and *C. revoluta* submerged immediately when encountering the PD, then sank to the bottom and entered the micropyle. The saccate pollen of *P. thunbergii* and *A. firma* submerged into the PD, but remained floating at the top and finally accumulated on the micropyle after PD withdrawal. In contrast, pollen of the angiosperms *P. suffruticosa*, *Salix babylonica*, and *O. violaceus* did not submerge, instead remaining clustered at the edge without entering the PD.

**Conclusions:**

We conclude that PD withdrawal is primarily determined by the dynamic balance between evaporation and ovule secretion, of which pollen is a critical stimulator. When conspecific pollen grains were submerged in the PD, ovule secretion was subsequently terminated and active absorption occurred. These processes cooperated to influence PD withdrawal. In addition, pollen grain behavior within PDs varied dramatically among taxa, and PDs played a role in distinguishing and transporting pollen in *G. biloba*.

## Background

How the female reproductive organ captures pollen is of fundamental importance for successful pollination of seed plants [1,2]. Most angiosperms are adapted to biotic pollination, in which pollen is delivered to stigmas by various animals. In contrast, the majority of gymnosperms are pollinated by means of wind (i.e., anemophily). Wind pollination was previously considered to be a random process. However, this pattern of pollination is now recognized as being highly coordinated and synchronous [1]. For example, several lines of evidence indicate that complex interactions may occur between pollen and the ovule [2]. Of these interactions, some gymnosperms may produce pollination drops (PDs) to capture airborne pollen grains and trigger their germination [3-5]. Therefore, in recent years, a great deal of attention has been paid to PD function.

PD production by ovules is part of the complex phenomena associated with pollination and fertilization [1], which occur in the phylogeny of plants as early as the pteridophytes [6]. Most gymnosperms produce PDs, except for some species of genera such as *AraucariaTsuga*, and *Saxegothaea*[1,4]. PDs are secreted from the nucellus [7] and play a key role in the pollination process. For example, PDs can be the landing site for pollen, and “scavenge” pollen grains from the air [8-12]. In addition, the major function of the PD is to transport pollen to the nucellar surface after PD withdrawal [3,4]. Thus, PD withdrawal is essential for the pollination of gymnosperms. Doyle and O’ Leary [13] first reported that the presence of pollen initiated rapid PD withdrawal in *Pinus*. Subsequently, several genera in which pollen-induced PD withdrawal has been observed have been used to show that pollen is the sole stimulus of PD withdrawal, e.g., *Phyllocladus*[9], *Acmopyle*[3], and *Juniperus*[5]. Nevertheless, in certain Podocarpaceae (Coniferales), the PD withdrawal process is merely physical and attributable to passive evaporation, independent of the presence of pollen [9]. In addition, Owens et al. [2] suggested that PD withdrawal signalled the end of active secretion and enabled rapid PD evaporation in *Chamaecyparis nootkatensis*. Thus, the questions of which factors influence PD withdrawal and how best to affect this process have been subjects of extensive controversy.

*Ginkgo biloba* L., usually described as a ‘living fossil’, is the only living representative of the order Ginkgoales. The species was distributed globally during the Jurassic [14] and survived in East Asia after the last ice age, but it is now cultivated and has been introduced into many other regions of the world. It is dioecious with an anemophilous pollination mode, and pollination and subsequent reproduction is highly efficient under natural conditions [15]. Several morphological and anatomical studies on *G. biloba* have shown that it has non-saccate pollen and a more or less upright ovule [6]. Its nucellar cells undergo programmed cell death to form a pollen chamber in which pollen germinates and develops pollen tubes [16], with the characteristic PD appearing on the micropyle during the pollination stage [17]. Taken together, these results show that the PD may play an important role in capturing and delivering pollen to the *G. biloba* ovule. However, no investigation of how PDs enhance pollination efficiency has been published to date. Moreover, the pollination mechanism of *G. biloba* is poorly documented, and little is known about the factors that influence PD withdrawal.

The purpose of the present study was to investigate PD withdrawal in *G. biloba* and to elucidate how the PD interacts with pollen to increase pollination efficiency. In addition, our results are discussed in comparison with other gymnosperms to reveal an ancient pollination pattern. Collectively, our findings provide a deeper insight into the wind-pollination mechanism of *G. biloba*.

## Methods

### Plant material

Healthy female *G. biloba* trees were selected at the Ginkgo Experimental Station in Yangzhou University, Yangzhou, China (32°20'N, 119°30'E). Materials were collected from 30 plants of similar age and height (~4 m) during the pollination period in early April 2009 and 2011.

### PD withdrawal

Branches with ovules that had just secreted PDs were collected and kept in the laboratory. Spurs (short shoots) with 3–5 ovules were removed with loppers and inserted into sponges immersed in trays of water. When PDs appeared, the following procedures were performed. (1) PD secretion was observed at 2-h intervals under a stereomicroscope (*n* = 50) under laboratory conditions (25°C and 50–70% relative humidity; RH). (2) Ovules were maintained in incubators (25°C and either 100% or ~50% RH), and the times of PD production and withdrawal were recorded (*n* > 30). (3) Sufficient quantities of PDs were collected in a centrifuge tube after they reached maximum volume. Then, 100 nL (about one PD volume) was transferred to a glass slide and subjected to various RHs (25°C and 0, 50–70% or 100% RH).

### Pollination

A human eyelash (paraffin-mounted on a length of wood) was used to collect pollen, which was gently applied onto PDs by slight contact [6]. Pollination drop withdrawal assays were performed as follows. (1) Viable conspecific and heterospecific gymnosperm pollen were collected soon after the pollen sac opened and stored in sterile vials. (2) Withdrawal of PDs without pollen deposition was examined (as a control) (*n* > 30). (3) Withdrawal of PDs pollinated with fresh pollen (10–20, 50–60, and >100 pollen grains, respectively). In addition, heat-treated (105°C for 12 h) and acetone-treated (8 h) pollen were examined (*n* > 30). (4) Withdrawal of PDs pollinated using fresh pollen (*n* = 50–60) of *Cycas revolutaPinus thunbergiiAbies firmaPaeonia suffruticosa*, and *Orychophragmus violaceus* was investigated (*n* > 30). (5) We placed ovules with PDs in the upright and inverted states and simultaneously pollinated their PDs (>100 pollen grains), observing PD withdrawal at 1-h intervals under a stereomicroscope. (6) PDs on short shoots were pollinated with pollen of *P. thunbergii* and *A. firma*. When the PDs had disappeared completely, the pollen chambers were stripped out and fixed in a solution containing 10% acetic acid and 90% ethanol. Fixed samples were hydrated, further softened with 1 M NaOH, and stained with 0.1% aniline blue (Sigma-Aldrich, St. Louis, MO, USA) for 10 min. Stained samples were observed using a Zeiss Axioplan microscope (Carl Zeiss Shanghai Co., Shanghai, China) equipped with an epifluorescence UV filter set (excitation filter at 365 nm, dichroic mirror at 395 nm, barrier filter long-pass at 420 nm).

### Determination of PD volume

A stereomicroscope with a micrometer eyepiece was used to measure the diameter of PDs at 1-h intervals from secretion to withdrawal (*n* > 30). The PD volume (*V*), which was assumed to be spherical, was calculated by the formula *V* = (4/3) π*r*^3^, where *r* is the radius.

### Observation of PD morphology

Morphological observations of PDs were carried out under a stereomicroscope (Motic SMZ-168-TL; Motic China Group Co. Ltd.), and digital images were captured using an Axiocam MRC camera (Carl Zeiss Shanghai Co.).

## Results

### PD formation and withdrawal

**Figure 1:**
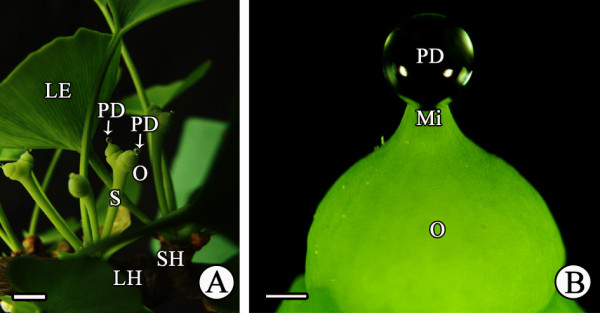
**PD formation in the field. (A)** PD production during pollination; **(B)** PD exuding from the micropyle. Le, leaf; Lh, long shoot; Mi, micropyle; O, ovule; PD, pollination drop; Sh, short shoot; S, stalk. Scale bars: A = 1 cm; B = 200 μm.

We investigated 2–4 pairs of *G. biloba* ovules at the apex of short shoots during pollination in field trees. The ovules are naked, without bracts, and more or less upright (Figure 1A). During pollination, the PD was secreted from the ovule and gradually formed on the micropyle; the PD was spherical with a diameter of 400–600 μm, i.e., about 2–3 times larger than the micropyle (Figure 1B). Subsequently, the PD withdrew and completely disappeared.

**Figure 2:**
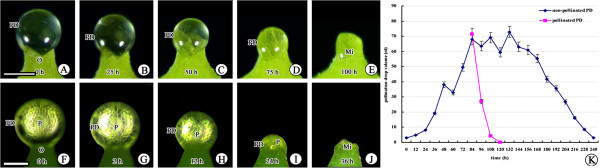
**PD withdrawal process. (A–E)** Nonpollinated PD withdraws slowly; **(F–J)** pollinated PD withdraws rapidly; **(K)** comparison of the withdrawal rates of non-pollinated and pollinated PDs. Mi, micropyle; O, ovule; P, pollen grains; PD, pollination drop. Scale bars: A–J = 200 μm.

Withdrawal of the PD is normally rapid and is not easily discerned in the field, thus we performed experiments under laboratory conditions (25°C and 50–70% RH) to observe, measure, and analyze the entire process of PD withdrawal. The PD is exposed from the micropyle. The PD volume was small but increased gradually after PD appearance, if not artificially pollinated. About 84 h later, the PD reached its maximum volume, which was maintained for about 48 h (Figure 2A). It then slowly decreased and disappeared within approximately 100 h (Figure 2A-E). Thus, PD appearance to its complete disappearance took ~240 h (Figure 2K). In contrast, when PDs of maximum volume were pollinated with fresh *G. biloba* pollen grains, they withdrew rapidly and completely disappeared within about 36 h (Figure 2F-K). Afterward, no further PD secretion was observed.

### Influence of ovule secretion and relative humidity on PD withdrawal

**Figure 3:**
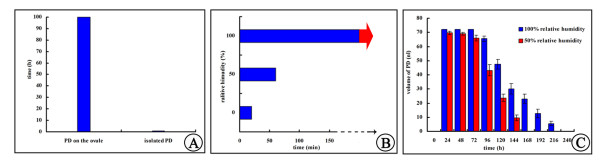
**PD withdrawal rate under different relative humidities. (A)** Comparison of the withdrawal rates of isolated PDs and PDs on ovules under laboratory conditions; **(B)** withdrawal rate under 50% and 100% relative humidity (red arrow, isolated PDs did not withdraw after 20 days under 100% relative humidity); **(C)** withdrawal rate under 50% and 100% relative humidity.

To understand better the factors that influence PD withdrawal, we first determined whether ovule secretion impacted PD withdrawal. We isolated PDs from ovules with a capillary tube when PD volume was at its maximum, placed a volume equivalent to one PD on a glass slide, and observed the PD withdrawal process. Under laboratory conditions (25°C and 50% RH), if not pollinated with fresh *G. biloba* pollen, PDs took only about 1 h to disappear, leaving some sticky and transparent material on the slide. However, PDs on living ovules maintained maximum volume for up to 100 h and then disappeared slowly (Figure 3A). These data suggest that the ovule itself slows down the PD withdrawal by secreting continuously when not pollinated, but the PD disappears completely because of evaporation.

Furthermore, we compared whether RH influenced PD withdrawal. PDs on glass slides were exposed to 25°C and RH of 0, 50%, or 100%. PDs took only about 20 min to completely disappear at 0% RH; however, the volume at 100% RH did not decrease for about 20 days (Figure 3B). Similar results were obtained for PDs on living ovules. In contrast to PD withdrawal under laboratory conditions (50–70% RH), which required about 100 h, complete PD withdrawal required approximately 240 h at 100% RH (Figure 3C). Thus, RH was an important factor in, and 100% RH significantly inhibited, PD disappearance.

### Influence of pollen quantity and viability on PD withdrawal

**Figure 4:**
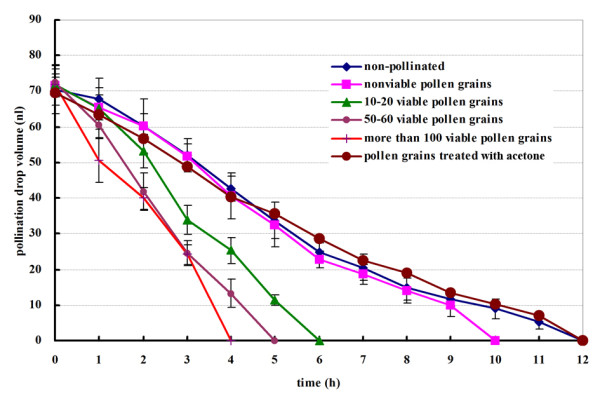
Influence of pollen quantity and viability on PD withdrawal.

We investigated the effect of pollen quantity and viability on PD withdrawal. We isolated ovules from short shoots, placed them on glass slides, and observed PD withdrawal under a stereomicroscope at 1-h intervals. PDs probably rapidly evaporated because of heat from the lamp; the volume of observed drops on isolated ovules under the microscope decreased much faster than for ovules on short shoots under laboratory conditions. In this experiment, the rate of decrease in PD volume was directly proportional to pollen quantity, i.e., the more pollen that was applied, the faster the drop withdrew. We also found that PDs pollinated with nonviable pollen grains withdrew faster than those not pollinated, but more slowly than those pollinated with fresh pollen grains; thus, nonviable pollen also caused a degree of PD withdrawal (Figure 4). However, acetone-treated pollen did not accelerate PD withdrawal, which was similar to the withdrawal rate of nonpollinated PDs.

### Influence of active absorption on PD withdrawal

**Figure 5:**
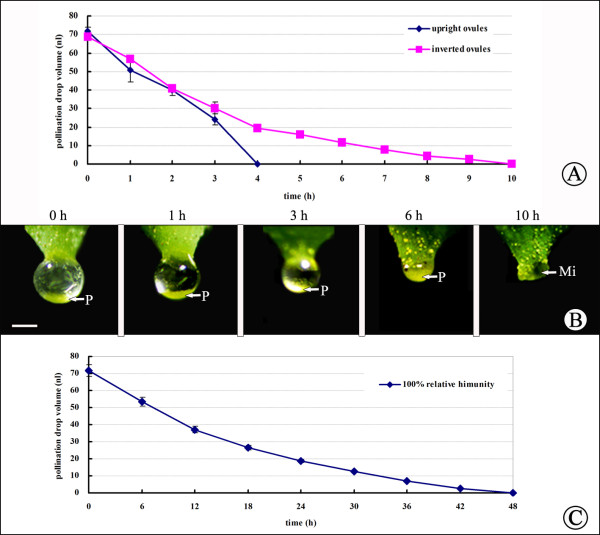
**Comparison of PD withdrawal in upright and inverted ovules. (A)** Comparison of PD withdrawal rates for upright and inverted ovules; **(B)** PD withdrawal of inverted ovules; **(C)** pollinated PD withdrawal under 100% relative humidity. O, ovule; P, pollen grains; Mi, micropyle. Scale bars = 200 μm.

To determine further whether active absorption plays a role in PD withdrawal, we placed ovules with PDs in the upright and inverted states. Upon pollination (>100 grains), PDs on upright ovules required about 4 h to withdraw completely under laboratory conditions (25°C, 50% RH) (Figure 4). However, pollen grains remained at the bottom of the PD of inverted ovules and required about 10 h to withdraw completely (Figure 5A, B). After withdrawal, no residue was visible on micropyles regardless of whether ovules were upright or inverted (Figure 5B). Furthermore, at 100% RH, PDs on ovules took 240 h (Figure 3C), whereas those on slides took about 20 days (480 h), to withdraw completely (Figure 3B). Importantly, once pollinated, PDs on ovules withdrew more rapidly and disappeared within 48 h, even at 100% RH (Figure 5C). These results suggest that active absorption by ovules may be involved in PD withdrawal.

### Influence of pollen from different species on PD withdrawal

**Figure 6:**
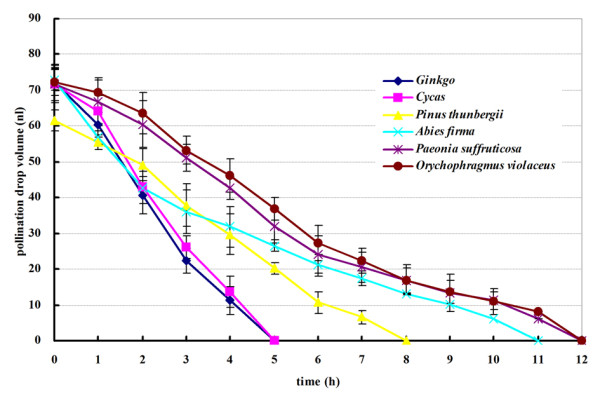
Influence of pollen from different species on PD withdrawal.

We next tested the influence of pollen of other species, including gymnosperms and angiosperms, on *G. biloba* PD. Pollination by different species exerted different effects on PD withdrawal (Figure 6). Pollen of *G. biloba* and *C. revoluta* showed a similar effect on PD withdrawal: PDs gradually withdrew within 5 h when pollinated by either species at the maximum PD volume (Figure 6). PDs also withdrew when pollinated by two gymnosperms, *P. thunbergii* and *A. firma*, although the rates were much lower; about 8 and 11 h, respectively, were required for complete PD withdrawal. We also applied the pollen of two angiosperms, *P. suffruticosa* and *O. violaceus*, to PDs. PDs required 12 h to withdraw and completely disappear after pollination by angiosperm pollen, similar to nonpollinated PDs, which implied that this pollen had little impact on PD withdrawal.

### Behavior of pollen from different species in PDs

**Figure 7:**
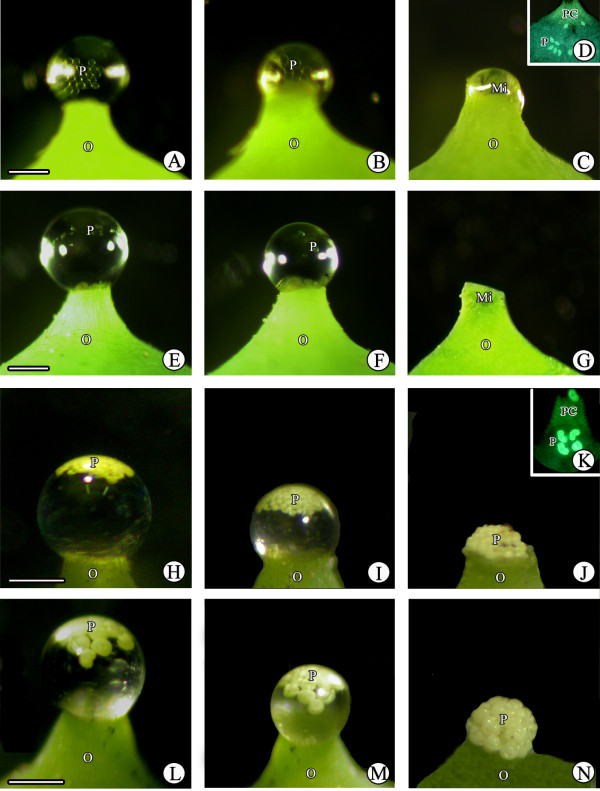
**Behavior of different gymnosperm pollen in the PD. (A–C)***Ginkgo biloba* pollen rapidly became submerged into the PD and entered the ovule upon PD withdrawal; **(D)***G. biloba* pollen grains in the pollen chamber. **(E–G)***Cycas revoluta* pollen also became submerged into the PD and entered the ovule. **(H–J)***Pinus thunbergii* pollen entered PDs, but did not sink into the PD, remaining afloat at the top; **(K)***P. thunbergii* pollen grains in the pollen chamber. **(L–N)***Abies firma* pollen could enter PDs, but remained afloat at the top. O, ovule; P, pollen grains; Mi, micropyle; PC, pollen chamber. Scale bars = 200 μm.

**Figure 8:**
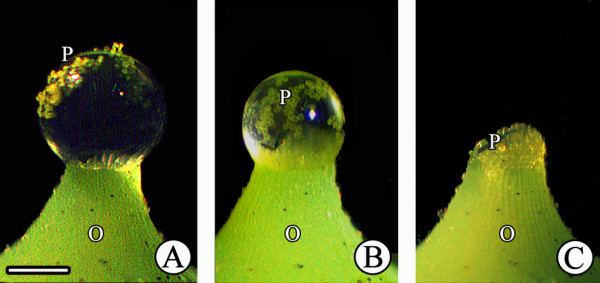
**Behavior of pollen from a wind-pollinated angiosperm in PDs. (A)** Pollen grains of *Salix babylonica* were applied to PDs; **(B)** pollen grains afloat on the surface; **(C)** pollen grains accumulated on the micropyle. O, ovule; P, pollen grains; Mi, micropyle. Scale bars = 200 μm.

**Figure 9:**
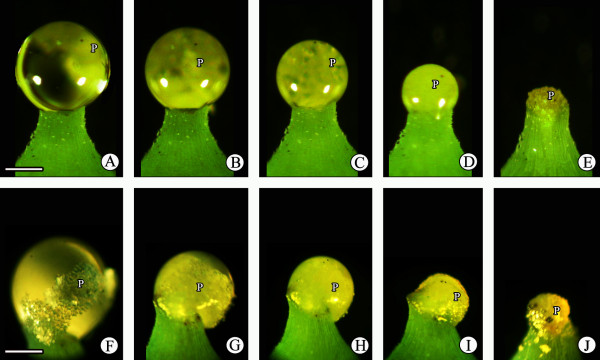
**Behavior of pollen from insect-pollinated angiosperms in PDs. (A–E)***Paeonia suffruticosa* and **(F-J)***Orychophragmus violaceus* pollen grains tended to clump together, remaining visible on the surface of PDs, and could not enter the ovule. O, ovule; P, pollen grains; Mi, micropyle. Scale bars = 200 μm.

Pollen of different species showed different behavior in *G. biloba* PDs. *Ginkgo biloba* pollen rapidly submerged into the PD after contacting the surface, then hydrated, swelled, and sank to the micropyle, finally entering the ovule upon PD withdrawal (Figure 7A-C). After PD withdrawal, *G. biloba* pollen was present in the pollen chamber (Figure 7D). *Cycas revoluta* pollen also submerged into the PD and entered the ovule, similar to that of *G. biloba* (Figure 7E-G). *Pinus thunbergii* and *A. firma* pollen also entered the PDs, although their saccate pollen is nonwettable. However, these pollen grains did not sink into the PD and remained floating at the top (Figure 7H, L), and, as a result, most grains accumulated only on the micropyle after complete PD withdrawal (Figure 7J, N). Furthermore, a few small *P. thunbergii* pollen grains were found in the pollen chamber (Figure 7K), whereas the larger pollen grains of *A. firma* could not enter the micropyle (Figure 7N). When the pollen grains of *S. babylonica*, a wind-pollinated angiosperm, were deposited onto *G. biloba* PDs, they remained afloat on the PD surface and did not submerge (Figure 8A, B). Thus, most *S. babylonica* pollen grains accumulated on the micropyle after PD withdrawal (Figure 8C). Similar to *S. babylonica*, *P. suffruticosa* and *O. violaceus* pollen grains (both insect-pollinated angiosperms) tended to clump together and remain visible on the PD surface (Figure 9A-E, F-J).

## Discussion

**Figure 10:**
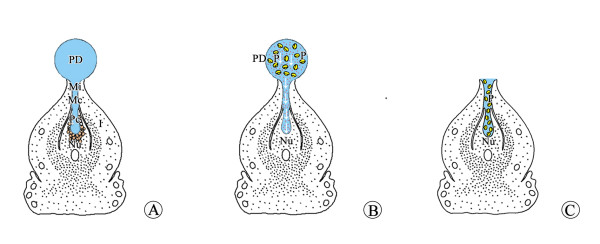
**Diagrammatic illustration of PD withdrawal. (A)** PD is produced constantly by nucellar tip cells and reaches its maximum volume at pollination. **(B)***Ginkgo biloba* pollen grains entered the PD, and the nucellar tip cells terminated secretion. **(C)** Pollen grains entered the pollen chamber along with PD withdrawal. I, integument; Mc, micropyle canal; Mi, micropyle; Nu, nucellus; P, pollen grains; Pc, pollen chamber; PD, pollination drop.

Withdrawal of the PD is affected by several factors, including RH, the quantity of viable pollen on the ovule, the state of ovule secretion, and the position of ovules [18-20]. Möller et al. [3] considered PD withdrawal to be an active process and that pollen can activate PD withdrawal and terminate PD secretion. Another explanation for PD withdrawal interprets it as a physical process attributed to passive evaporation rather than active withdrawal, i.e., independent of the presence of pollen [8,9,21]. In this study, we found that PDs withdrew quickly either at low humidity or when pollinated with a large quantity of viable conspecific pollen. In addition, when PDs were isolated from ovules, nonpollinated PDs took 1 h to withdraw completely, whereas the disappearance of nonpollinated PDs on the living ovule required over 100 h, which suggested that the ovule could secrete the PD continuously during pollination. These results indicate that PD withdrawal was determined by the balance between evaporation and ovule secretion, in which pollen was an important stimulus (Figure 10).

When not pollinated, PDs on ovules disappeared at 100% RH within 240 h (12 days)—less than PDs on slides, which took about 20 days to withdraw completely. Once pollinated, PD secretion was inhibited and PDs on ovules disappeared within 48 h, even at 100% RH. In addition, complete withdrawal of PDs on inverted ovules required more time (~10 h) than those on upright ovules (~4 h). Regardless of the upright or inverted state of ovules, PDs completely disappeared without leaving a sticky residue on micropyles. These results suggest that an active absorption process was involved in PD withdrawal.

The effect of foreign pollen on PD withdrawal can be interpreted in several ways. Möller et al. [3] observed that foreign pollen triggered PD retraction, but with a lesser stimulatory effect than conspecific pollen, in *Phyllocladus trichomanoides* D. Don and *Phyllocladus toatoa* Molloy. Tomlinson et al. [9] showed that in *Phyllocladus* foreign pollen initiated drop retraction, presumably via a biochemically based mechanism [5,22]. In the present study, we also found that PD withdrawal was triggered by foreign pollen, but that the effect varied among taxa. Pollen from *C. revoluta*, a gymnosperm and the taxon most closely related to *G. biloba*[23] among the study taxa, induced more rapid PD withdrawal compared to that of conifers, represented by *P. thunbergii* and *A. firma*. However, pollen from the angiosperms *P. suffruticosa* and *O. violaceus* had little impact on PD withdrawal, which was similar to nonpollinated PDs. These results indicated foreign pollen from gymnosperms and angiosperms had different impacts on the pollination mechanism, and only pollen that enters the conspecific PDs can trigger their withdrawal.

PD is not only a landing site, but also a germination medium in which pollen can be transported to the nucellus surface upon PD withdrawal. Tomlinson et al. [9] found that pollen from different species was collected by the PDs of podocarps under normal conditions. Some other reports suggest that pollen selection may occur when foreign pollen encounters PDs, and thus PDs may represent a direct method for female selection [1]. In this study, PDs of *G. biloba* accepted and transported their own pollen into the pollen chamber by their withdrawal. In addition, we observed that *C. revoluta* pollen rapidly sank to the bottom after landing on the PD. In contrast, *Pinus* pollen always floated and accumulated on the micropyle after PD withdrawal, although it entered the *G. biloba* PD. In contrast, pollen of the wind-pollinated angiosperm *S. babylonica* and the entomophilic angiosperms *P. suffruticosa* and *O. violaceus* could not enter the *G. biloba* PD and instead remained on the surface. These results indicate that pollen behavior within PDs varied dramatically among taxa and that the PD is involved in the regulation of pollen behavior. Therefore, such different behavior may provide evidence for interspecific relationships in terms of PDs.

## Conclusions

We investigated the PD withdrawal process and analyzed the PD withdrawal mechanism of *G. biloba*. This study has three main findings: (1) when the PD was pollinated with conspecific pollen, ovule secretion was terminated, thus PD size gradually decreased because the evaporation rate exceeded the active secretion rate; (2) active absorption accelerated PD withdrawal after pollen stimulation; and (3) PD withdrawal was triggered also by foreign pollen, but the precise effect and behavior varied among taxa. Only the pollen of closely related species was liable to become submerged and trigger PD withdrawal. We conclude that PD withdrawal in *G. biloba* represents a dynamic balance between evaporation and ovule secretion, that active absorption is involved in PD withdrawal, and that the PD has a function in the regulation of pollen behavior. Our study provides novel data on PD withdrawal during pollination processes and adds to our understanding of the function of PDs in accepting and transporting pollen into the ovule.

## Authors’ contributions

LW and BJ designed the project; LZ, YL and DW performed the SEM and TEM observations and semi-thin sections experiment; XXJ and ZM were involved in cultivating ovules and collecting and measuring PDs; BJ drafted the manuscript. All authors read and approved the final manuscript.
